# Effects of Allergic Predisposition on Corneal Biomechanical Properties in Normal Corneas: A Retrospective Study

**DOI:** 10.14789/ejmj.JMJ24-0028-OA

**Published:** 2025-01-30

**Authors:** SAKYO KANEHARA, MASAHIRO YAMAGUCHI, YOSHIMUNE HIRATSUKA, SHIRO AMANO, NOBUYUKI EBIHARA

**Affiliations:** 1 Juntendo University Graduate School of Medicine, Tokyo, Japan; 1 Juntendo University Graduate School of Medicine, Tokyo, Japan; 2 Department of Ophthalmology, Juntendo University Urayasu Hospital, Chiba, Japan; 2 Department of Ophthalmology, Juntendo University Urayasu Hospital, Chiba, Japan; 3 Inouye Eye Hospital, Tokyo, Japan; 3 Inouye Eye Hospital, Tokyo, Japan

**Keywords:** allergic predisposition, corneal biomechanical properties, keratoconus

## Abstract

**Objective:**

Pathogenic factors that reduce corneal biomechanical properties (CBPs) remain unclear. This study aimed to retrospectively analyze the effect of allergic predisposition on CBPs in normal corneas.

**Design:**

Retrospective study.

**Methods:**

Patients with atopic dermatitis (8 eyes), allergic conjunctivitis (18 eyes), vernal conjunctivitis (5 eyes), and atopic keratoconjunctivitis (14 eyes) in their 10s-30s who visited the Juntendo University Hospital were considered to have an allergic predisposition (+). Additionally, 32 eyes were included in the healthy control group without allergic predisposition (-). CBP parameters (Corvis Biomechanical Index, deformation amplitude [DA], peak distance, A1 velocity, and A2 velocity [A2V]) were assessed using Corvis ST (Oculus, Wetzlar, Germany) in all patients. The relationship between the CBP parameters and allergic predisposition was evaluated using multiple linear regression analysis with generalized estimating equations.

**Results:**

Corneal shape analysis using CASIA (Tomey Corp., Nagoya, Japan) confirmed that no complications associated with keratoconus were present. Multivariate analysis revealed that an allergic predisposition (+) had a strong influence on A2V (*p* < 0.001) and that DA was affected by allergic predisposition (*p* = 0.002).

**Conclusions:**

Allergic predisposition may affect CBP parameters in normal corneas, suggesting that A2V may be an early marker of keratoconus onset.

## Introduction

Keratoconus is a progressive binocular disease characterized by the thinning and anterior protrusion of the central and paracentral portions of the cornea, resulting in strong myopic and irregular astigmatism^1-3)^. Mild cases may present no subjective symptoms; however, strong astigmatism leads to poor spectacle-corrected visual acuity in moderate cases, requiring rigid gas-permeable contact lenses (RGPCLs) for correction. If the condition progresses, corneal crosslinking or intracorneal ring placement are possible treatment modalities. In severe cases, rupture of the Descemet membrane may occur, resulting in transient acute corneal edema and vision loss. Although acute corneal edema often resolves spontaneously within a few months, corneal scarring follows. Patients who have difficulty correcting their vision with RGPCLs, including those with scarring after acute edema or those who have difficulty wearing such lenses, may require deep anterior lamellar keratoplasty or penetrating keratoplasty. Despite keratoconus being considered to have a good prognosis after corneal transplantation, complications such as rejection, endothelial dysfunction, suture infection, wound dehiscence, and protrusion recurrence can occur. Therefore, accurately diagnosing keratoconus at an early stage, leading to early treatment, is important. However, the pathogenesis of keratoconus remains not fully understood.

Complications of allergic conjunctivitis are knowingly a risk factor for keratoconus. Although keratoconus was initially thought to be a noninflammatory corneal dilatation, recent reports have suggested that inflammatory mediators may be involved in its development and progression^4-9)^.

Corneal biomechanical properties (CBPs) refer to the ease of deformation of the cornea when a load is applied to it. The following four points are significant for this measurement: (1) Understanding the correct intraocular pressure (IOP), as the thicker the cornea or the smaller the radius of the corneal curvature, the higher the IOP will be measured; (2) examining the impact of refractive surgery on safety and prediction accuracy; (3) predicting keratoconus onset and corneal ectasia; and (4) evaluating the effectiveness of corneal cross-linking. CBP data obtained from Corvis ST (Oculus, Wetzlar, Germany) have been applied in the fields of corneal diseases and glaucoma; however, few reports in the field of allergic diseases using such data have been published.

CBPs are reduced before corneal protrusion occurs^10)^. A large absolute value of A2 velocity (A2V), one of the CBPs, indicates a soft cornea. Keratoconic eyes possess higher A2V values than normal eyes^11)^. If an allergic predisposition affects CBPs, including the A2V levels, it is possible that such an effect reflects the early changes occurring in the normal eye as it progresses to keratoconus. Therefore, this study aimed to evaluate the influence of allergic predisposition on the CBPs of normal corneas. We found that allergic predisposition had a strong influence on A2V. Therefore, allergic predisposition may affect CBP parameters in normal corneas, suggesting that A2V may be an early marker of keratoconus onset.

## Materials and Methods

### Ethical considerations

Patient consent was waived due to the retrospective design of the study. The study was conducted in accordance with the Declaration of Helsinki, and the protocol was approved by the Institutional Review Board of Juntendo University (protocol code: E21-0141; date of approval: October 8, 2021).

### Study design and population

This retrospective study included patients in their 10s-30s who visited the Ophthalmology Department of the Juntendo University Hospital. As keratoconus is thought to develop in the mid-teens and to stop progressing around the age of 25-30 years, the research subjects were eyes that did not develop keratoconus in this age group. Patients with atopic dermatitis (8 eyes), allergic conjunctivitis (18 eyes), vernal conjunctivitis (5 eyes), or atopic keratoconjunctivitis (14 eyes) were defined as having an allergic predisposition. Additionally, 32 eyes without allergic predisposition were included in the healthy control group. Because normal eyes were the subjects of the study, eyes with abnormal corneal shapes, including eyes with keratoconus, were excluded. In patients with keratoconus in only one eye, the eye without abnormal corneal shape is called “forme fruste keratoconus” (FFK). After one eye is diagnosed with keratoconus, 40% and 50% of FFK cases progress to keratoconus within 6 years^1)^ and within 16 years^2)^, respectively. Therefore, eyes with suspected FFK were excluded from the study.

### Data collection

**Figure 1:**
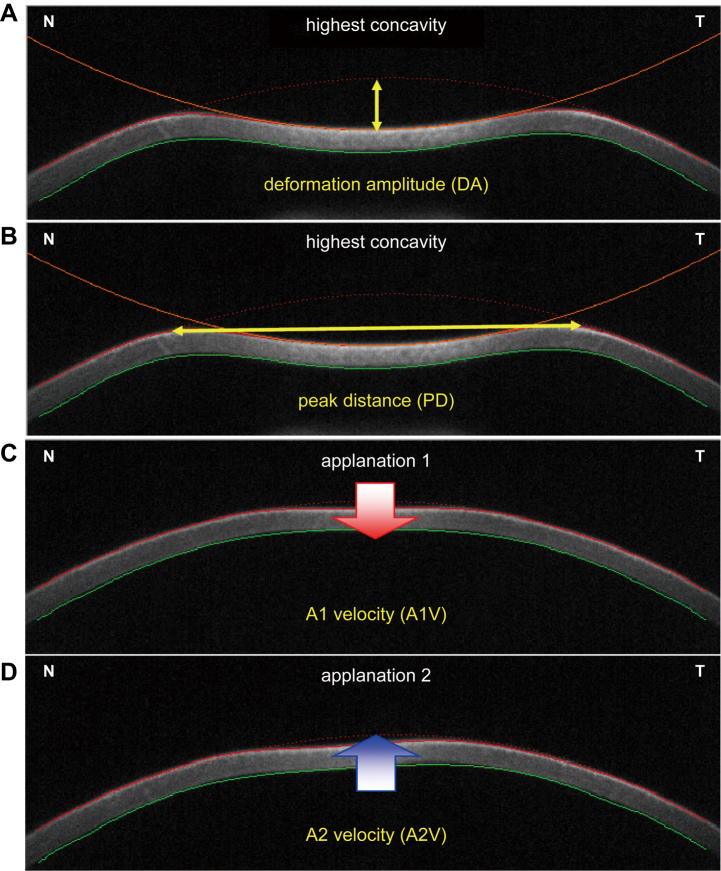
Schematic diagram of CBP parameters obtained from Corvis ST. When air is blown onto the cornea, the cornea deforms in the following order: initial state, applanation 1, highest concavity, applanation 2, and back to the initial state. (A) DA refers to the maximum deformation amplitude at the apex of the cornea from the initiation of the air puff to the highest concavity. (B) PD is defined as the distance between the peaks at the highest concavity, calculated using Scheimpflug imaging. (C and D) A1V is the inward (vitreous side) corneal deformation velocity in the first flattened state (applanation 1) after air emission, whereas A2V is the outward corneal deformation velocity in the second flattened state (applanation 2). CBP, corneal biomechanical properties; DA, deformation amplitude; PD, peak distance; A1V, A1 velocity; A2V, A2 velocity; N, nasal; T, temporal.

The CBP parameters were assessed in all patients using Corvis ST (Oculus, Wetzlar, Germany). Corvis ST measures corneal deformation by blowing air on the cornea, acquiring Scheimpflug imaging during corneal movement, and measuring parameters of CBPs and IOP. Seven parameters were selected for evaluation: 1) IOP; 2) central corneal thickness (CCT); 3) Corvis Biomechanical Index (CBI); 4) deformation amplitude (DA); 5) peak distance (PD); 6) A1 velocity (A1V); and 7) A2V. The CBI is a comprehensive parameter derived from six elements: DA (1 mm), DA (2 mm), A1V, standard deviation of the DA at the highest concavity, Ambrosio relational thickness to the horizontal profile, and stiffness. It can be used to determine the risk of keratoconus and corneal ectasia, with values close to zero in healthy eyes and values close to 1 for eyes with keratoconus and corneal ectasia. DA refers to the maximum deformation amplitude at the apex of the cornea from the initiation of the air puff to the highest concavity. PD is the distance between the peaks at the highest concavity, calculated using Scheimpflug imaging. A1V is the inward (vitreous side) corneal deformation velocity in the first flattened state after air emission, whereas A2V is the outward corneal deformation velocity in the second flattened state (Figure 1). Corneal morphology analysis was performed using CASIA (Tomey Corp., Nagoya, Japan) to confirm the absence of complications associated with keratoconus.

### Statistical analysis

First, we described the characteristics of the study participants. The averages of continuous variables, including age, IOP, and corneal thickness, were compared using a t-test. The relationship between the CBP parameters (CBI, DA, PD, A1V, and A2V) captured by Corvis ST and allergic predisposition was analyzed. Linear regression and multiple linear regression analyses with generalized estimating equations to control for inter-eye correlation were performed. Initially, we calculated the coefficients and 95% confidence intervals (CIs). The coefficients with 95% CIs after adjustment for allergic predisposition, IOP, CCT, and age in the multivariate analysis were then estimated. Significant differences were defined as those with *p* < 0.05 and *p* < 0.001. All statistical analyses were performed using Stata version 15.0 (StataCorp LLC, College Station, TX, USA).

## Results

### Patient characteristics

The patient characteristics are shown in Table 1. Twenty-four patients and 32 eyes comprised the group without allergic predisposition, whereas the group with allergic predisposition included 24 patients and 45 eyes (atopic dermatitis, *n* = 8; allergic conjunctivitis, *n* = 18; vernal conjunctivitis, *n* = 5; and atopic keratoconjunctivitis, *n* = 14). Although age and IOP did not differ significantly between the groups with and without allergic predisposition (*p* = 0.19 and *p* = 0.13, respectively), CCT was significantly lower in the allergic predisposition group than in the non-allergic predisposition group (*p* < 0.001).

**Table 1:** Patient characteristics

	Allergic predisposition (-)	Allergic predisposition (+)	*p*-value
Cases/eyes	24/32	24/45	
Age (years)	30.7 ± 8.0	28.4 ± 7.2	0.19
IOP (mmHg)	15.5 ± 2.4	16.4 ± 2.6	0.13
CCT (μm)	550.3 ± 27.7	520.3 ± 29.2	<0.001**

**p < 0.001 was considered to indicate a significant difference. IOP, intraocular pressure; CCT, central corneal thickness.

### Linear regression analysis

Table 2 shows the results of the univariate linear regression analysis with generalized estimating equations. CBI was affected by CCT and allergic predisposition (*p* < 0.001 and 0.003, respectively). DA was affected by age, IOP, and allergic predisposition (*p* = 0.002, *p* < 0.001, and *p* = 0.01, respectively) but not by CCT (*p* = 0.52). PD was less affected by age, IOP, CCT, and allergic predisposition (*p* = 0.84, *p* = 0.32, *p* = 0.92, and *p* = 0.52, respectively). Similarly, A1V was less affected by these factors (*p* = 0.18, *p* = 0.88, *p* = 0.45, and *p* = 0.45, respectively). A2V was affected by age and allergic predisposition (*p* = 0.001 and *p* < 0.001, respectively), but not by IOP or CCT (*p* = 0.31 and *p* = 0.12, respectively).

**Table 2:** Results of the univariate regression analysis

Factor		Coefficient	*p*-value	95% CI
CBI				
	Age (+1 year)	0.006	0.25	-0.004 to 0.169
	IOP (mmHg)	-0.012	0.30	-0.034 to 0.010
	CCT (μm)	-0.007	<0.001**	-0.009 to -0.005
	Allergic predisposition	0.2716	0.003*	0.094 to 0.449
DA				
	Age (+1 year)	0.004	0.002*	0.002 to 0.007
	IOP (mmHg)	-0.03	<0.001**	-0.036 to -0.024
	CCT (μm)	-0.0002	0.52	-0.001 to 0.001
	Allergic predisposition	-0.07	0.01*	-0.123 to -0.015
PD				
	Age (+1 year)	-0.0008	0.84	-0.008 to 0.008
	IOP (mmHg)	0.008	0.32	-0.008 to 0.023
	CCT (μm)	0.00008	0.92	-0.002 to 0.002
	Allergic predisposition	0.041	0.52	-0.084 to 0.165
A1V				
	Age (+1 year)	-0.0004	0.18	-0.0009 to 0.0002
	IOP (mmHg)	0.0002	0.88	-0.002 to 0.002
	CCT (μm)	-0.00005	0.45	-0.0001 to 0.00007
	Allergic predisposition	0.003	0.45	-0.005 to 0.012
A2V				
	Age (+1 year)	0.004	0.001*	0.002 to 0.007
	IOP (mmHg)	-0.003	0.31	-0.007 to 0.002
	CCT (μm)	0.0006	0.12	-0.0001 to 0.001
	Allergic predisposition	-0.096	<0.001**	-0.139 to -0.054

*p < 0.05 and **p < 0.001 were considered to indicate a significant difference. CBI, Corvis Biomechanical Index, DA, deformation amplitude; PD, peak distance; A1V, A1 velocity; A2V, A2 velocity; CI, confidence interval; IOP, intraocular pressure; CCT, central corneal thickness.

### Multiple linear regression analysis

Table 3 presents the results of the multiple linear regression analysis with the generalized estimating equations. Multivariate analysis was performed with allergic predisposition, IOP, CCT, and age as adjustment factors. The CBI was only affected by CCT (*p* < 0.001). The DA was affected by IOP and allergic predisposition (*p* < 0.001 and 0.002, respectively). No associations were found between any of the factors (age, IOP, CCT, or allergic predisposition) and PD. The results also indicated no association between any of the aforementioned factors and A1V. A2V was significantly associated with allergic predisposition (*p* < 0.001).

**Table 3:** Results of the multivariate regression analysis

Factor		Coefficient	*p*-value	95% CI
CBI				
	Age (+1 year)	0.0004	0.95	-0.011 to 0.011
	IOP (mmHg)	-0.018	0.07	-0.037 to 0.001
	CCT (μm)	-0.007	<0.001**	-0.009 to -0.005
	Allergic predisposition	-0.019	0.78	-0.153 to 0.116
DA				
	Age (+1 year)	0.002	0.10	-0.0003 to 0.003
	IOP (mmHg)	-0.028	<0.001**	-0.034 to -0.002
	CCT (μm)	-0.0006	0.06	-0.001 to 0.00002
	Allergic predisposition	-0.052	0.002*	-0.084 to -0.019
PD				
	Age (+1 year)	0.00009	0.98	-0.008 to 0.008
	IOP (mmHg)	0.006	0.42	-0.009 to 0.021
	CCT (μm)	0.0004	0.70	-0.001 to 0.002
	Allergic predisposition	0.045	0.53	-0.098 to 0.187
A1V				
	Age (+1 year)	-0.0003	0.24	-0.0009 to 0.0002
	IOP (mmHg)	-0.0001	0.92	-0.002 to 0.002
	CCT (μm)	-0.00003	0.67	-0.0002 to 0.0001
	Allergic predisposition	0.0026	0.74	-0.008 to 0.010
A2V				
	Age (+1 year)	0.003	0.001*	0.001 to 0.005
	IOP (mmHg)	0.0002	0.917	-0.004 to 0.004
	CCT (μm)	0.00003	0.924	-0.0006 to 0.0006
	Allergic predisposition	-0.089	<0.001**	-0.129 to -0.048

*p < 0.05 and **p < 0.001 were considered to indicate a significant difference. The *p*-values for the effects of age, IOP, central corneal thickness, and allergic predisposition on various Corvis ST parameters (CBI, DA, PD, A1V, and A2V) are shown. CBI, Corvis Biomechanical Index, DA, deformation amplitude; PD, peak distance; A1V, A1 velocity; A2V, A2 velocity; CI, confidence interval; IOP, intraocular pressure; CCT, central corneal thickness.

## Discussion

No difference in age or IOP was found between the allergic and non-allergic predisposition groups; however, significant thinning of the CCT was identified in the participants with an allergic predisposition (Table 1). Patients with allergic conjunctivitis and atopic keratoconjunctivitis have a thinner CCT than healthy individuals, with values of approximately 520 μm, similar to those in the present study^12, 13)^.

Wang et al.^8)^ found that the tomographic biomechanical index (TBI) was significantly higher in the allergic conjunctivitis group than in non-allergic conjunctivitis group, suggesting that TBI is a function of the CBPs. In their report, no significant difference in CBI was found between patients with and without allergic conjunctivitis (*p* = 0.15). As shown in Table 2, CCT and allergic predisposition affected CBI in this study according to the univariate analysis. However, because CBI is affected by age, IOP, and CCT^14)^, multivariate analysis with adjustment for these factors was necessary, which showed that allergic predisposition did not affect CBI values (Table 3). Additionally, because CASIA, rather than Pentacam, was used to perform the corneal morphological analysis in this study, the TBI values were not calculated and could not be compared with those in previous reports. In the present study, patients with atopic dermatitis, allergic conjunctivitis, vernal conjunctivitis, and atopic keratoconjunctivitis were categorized into the allergic predisposition group, whereas a previous report by Wang et al.^8)^ included only patients with allergic conjunctivitis. The results of our univariate analysis differed from those of this previous report, possibly owing to differences in the patients’ comorbidities. The CBI in normal corneas was unaffected by allergic predisposition. However, multivariate analysis of the effect of allergic predisposition on the parameters of CBPs in eyes with keratoconus showed that allergic predisposition significantly affected CBI (*p* = 0.011) (unpublished data). These results suggest that CBI is effective in detecting keratoconus after its onset but is not suitable for detecting decreased parameters of CBPs in normal corneas before its onset.

The A2V values found in the present study differed from those in previous reports. Multivariate analysis (Table 3) showed that allergic predisposition had no effect on A1V but had a strong effect on A2V. Elham et al.^11)^ reported that the A2V was higher in eyes with keratoconus than in healthy eyes and that A2V corrected for CCT distinguished between healthy and keratoconus eyes with a sensitivity of 75% and a specificity of 75%. Because eyes in the early stage of keratoconus change from a posterior corneal shape, the A2V value, which is the outgoing corneal deformation velocity when the corneal shape is restored from the highest concavity, may change first. Therefore, an allergic predisposition in normal corneas may affect the A2V value, which may be a marker for keratoconus onset. Allergic predisposition affecting A2V values has not yet been reported; thus, we believe that our study presents a novel finding. For patients with an allergic predisposition, if the A2V increases progressively with examinations, early detection and treatment of keratoconus may reduce the risk of developing complications related to the disease. However, because a multivariate analysis of A2V with adjustment for age, IOP, and CCT is needed, the items of the modified A2V adjusted for these factors are required.

This study had some limitations, which should be acknowledged. Because the number of cases of each allergic disease was small, all patients with atopic dermatitis, allergic conjunctivitis, vernal conjunctivitis, and atopic keratoconjunctivitis were considered to possess an allergic predisposition. Accumulating more cases of each allergic disease to subdivide and reanalyze the data for each disease is warranted in the future. We also plan to investigate how differences in the severity of allergies and treatment details affect CBP in future studies. Multivariate analysis indicated that allergic predisposition may affect A2V values and may be a useful marker for the development of keratoconus. However, we could not explain the specific molecular and cellular mechanisms by which allergic predisposition affects A2V. Therefore, in the future, we will investigate whether A2V and CBI were correlated with the expression levels of cytokines, chemokines, and lipids in the tear fluid of healthy and allergic eyes.

Our multivariate analysis showed that allergic predisposition affected the parameters of CBPs in normal corneas, and the results of this study indicated that allergic predisposition is not only an aggravating factor for keratoconus but also one of the factors for its onset.

## Funding

The authors received no financial support for the research.

## Author contributions

Conceptualization, SK; methodology, SK; validation, SK and MY; formal analysis, YH; investigation, SK and MY; data curation, SK and MY; writing - original draft preparation, SK and MY; writing - review & editing, YH, SA, and NE; visualization, SK; supervision, NE; project administration, NE. All authors have read and approved the final version of the manuscript.

## Conflicts of interest statement

The authors declare that there are no conflicts of interest.

## Data availability statement

The datasets used in this study are available from the corresponding author upon request.
